# Multi-Channel Vibration Signal Analysis for Flexible Bearing Fault Diagnosis of Industrial Robot Harmonic Drives

**DOI:** 10.3390/s26072134

**Published:** 2026-03-30

**Authors:** Rongzhou Lin, Xiaohui Duan, Tongxin Gao

**Affiliations:** 1 School of Mechanical Engineering, University of Science and Technology Beijing, Beijing 100083, China; 2 China North Vehicle Research Institute, Beijing 100072, China

**Keywords:** industrial robot, fault diagnosis, multi-channel signal, independent component analysis

## Abstract

In industrial robots, harmonic drive flexible bearings are prone to faults, and fault diagnosis is essential for preventing unexpected downtime. However, vibration signals acquired from robot joints are often non-stationary and contaminated by strong multi-source interference, including motion-induced interference and vibrations induced by the deformation of flexible components. Such interference severely masks the subtle signatures of faults. To address this issue, this paper proposes a fault diagnosis framework that leverages multi-channel vibration signals to enhance fault-related features. First, angular resampling is applied to eliminate speed-induced non-stationarity. Second, envelope extraction is utilized to obtain demodulated signals suitable for independent component analysis (ICA). Subsequently, ICA is employed to extract fault-related components from the multi-channel signals. Finally, the fault-related independent component is identified and analyzed via envelope order spectrum analysis. Experimental validation on an industrial robot under both single-joint and multi-joint operating conditions demonstrates the effectiveness of the proposed framework. The method suppresses multi-source interference and achieves accurate fault diagnosis for flexible bearings under complex operating conditions, with quantitative validation confirming the diagnostic performance of the proposed framework.

## 1. Introduction

Industrial robots are core equipment in intelligent manufacturing, and their reliability is essential for stable production. Harmonic drives are widely used in robot joints due to high precision and reduction ratio. However, the flexible thin-walled rolling bearing in harmonic drives suffers continuous cyclic deformation and is prone to faults. Once a fault manifests in harmonic drive flexible rolling bearings, it will result in the degradation of motion accuracy and the risk of unexpected shutdown. Therefore, flexible rolling bearing fault diagnosis is crucial for the reliable operation of robots.

Extensive research has been conducted on the fault diagnosis of flexible bearings in harmonic drives. Feng et al. [1] established vibration signal analytic models of flexible bearing faults. Yu et al. [2] proposed a Gini-index-based time-reassigned multi-synchrosqueezing transform to extract the instantaneous fault frequency of flexible bearing faults. By optimizing the parameters of the Morlet wavelet transform, Yu and Zhao [3] successfully extracted and reconstructed the hidden fault characteristic impulses from the original signal.

However, extracting fault features of flexible bearings from robotic vibration signals remains a significant challenge. First, unlike stationary machinery, robots operate under variable speeds. Time-varying rotational speed causes continuous drift of fault characteristic frequencies, leading to severe spectral smearing in conventional Fourier analysis and preventing fault energy from concentrating at discrete spectral peaks [4]. Second, even if stationarity is restored, the signal remains heavily contaminated. The harmonic drive inherently generates strong, high-energy vibrations, primarily arising from inherent structural resonances, the elastic deformation of the flexspline, and rotational harmonic vibrations [3,5]. Furthermore, the accelerometer captures coupled vibrations from adjacent joints [6]. These interferences mask the weak fault impulses, rendering standard frequency analysis ineffective.

In the broader field of condition monitoring, diverse strategies have been explored to tackle such complexities. For instance, cage instantaneous angular speed combined with acoustic emission has been employed to detect incipient bearing anomalies [7], while accelerometric-signal-based monitoring of torque/speed equilibrium points has been validated in aircraft hybrid electric propulsion systems [8]. To address the specific complexity of vibration signals under variable speeds, sparse multi-variate time series models [9] and dynamic time-varying response modules [10] have been applied to gearboxes and gas turbine bearings. Moreover, multi-channel signals have been proven to possess significant potential for fault diagnosis in robot joint scenarios. Guo et al. [11] and Luan et al. [12] demonstrated the effectiveness of multi-channel sensor fusion. Building on this, researchers like Yang et al. [13,14] and Ribeiro et al. [15] employed deep learning techniques integrated with multi-channel signals, and advanced self-supervised models have been proposed to mitigate reliance on labeled data [16,17]. However, these data-driven methods fundamentally act as black boxes and require extensive data for training. As a result, it is challenging to apply them effectively in complex industrial scenarios where an intuitive understanding of fault mechanisms is required.

To isolate fault features from this complex mixture with physical interpretability, blind source separation (BSS) techniques, particularly independent component analysis (ICA) [18], offer a promising solution by leveraging the statistical independence of multi-channel signals. For instance, Mika et al. [19] successfully applied linear ICA algorithms to separate overlapping vibration sources and enhance localized defect features in rolling bearings. However, the application of ICA faces fundamental challenges. First, the fault features in different channels are modulated into distinct frequency bands, preventing the direct application of standard ICA for separation [20]. Second, the inherent ambiguities in amplitude and permutation make quantitative fault assessment difficult [21,22,23]. Existing methods either lack physical interpretability or fail to simultaneously address variable-speed non-stationarity and strong multi-source interference in robot joint scenarios, leaving mechanism-based diagnosis of harmonic drive flexible bearings an open challenge.

To address this, this paper proposes a diagnostic framework that leverages the spatial diversity of multi-channel vibration signals to separate fault features from strong multi-source interference under variable-speed operating conditions, integrating angular resampling, envelope extraction and ICA. The framework addresses the dual challenges of non-stationarity and multi-source interference. The main contributions of this paper are summarized as follows:

1. The multi-source interference mechanisms in robot joint vibration signals are systematically analyzed, including motion-induced kinematic interference and cross-joint structural coupling;
2. A multi-channel vibration signal processing framework integrating angular resampling, envelope extraction, and ICA is proposed to address variable-speed non-stationarity and multi-source interference in robot joints;
3. Mechanism-based fault diagnosis is achieved for harmonic drive flexible bearings under strong multi-source interference, with effectiveness validated under both single-joint and multi-joint operating conditions.

The remaining content of the paper is organized as follows. Section 2 establishes the theoretical signal models and Section 3 analyzes the vibration characteristics under variable speed and interference. Section 4 details the proposed feature extraction framework based on angular resampling, envelope extraction, and ICA. Section 5 presents the experimental verification and result analysis. Finally, Section 6 concludes the paper.

## 2. Fault Vibration Signal Analytical Model of Flexible Bearing

**Figure 1:**
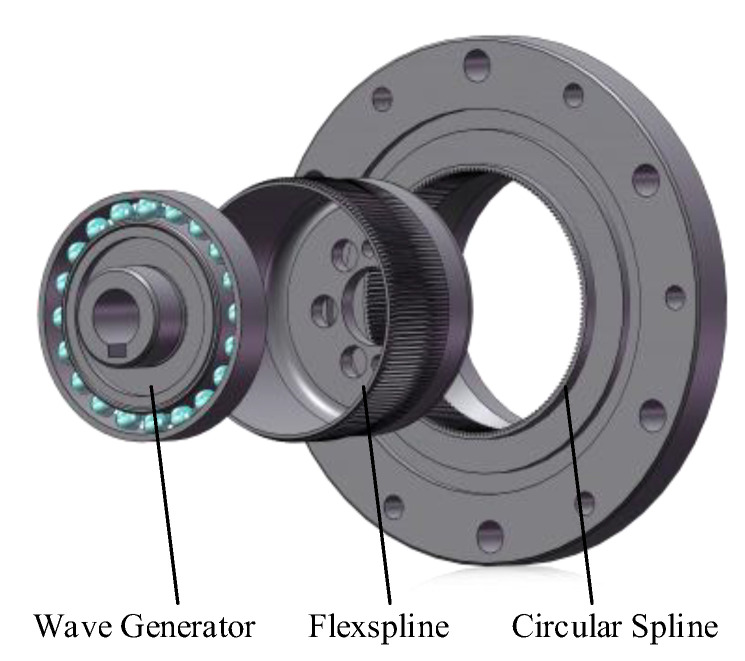
Main components of a harmonic drive.

A harmonic drive consists of a wave generator (incorporating a flexible bearing), a flexspline, and a rigid circular spline, as shown in Figure 1. A localized fault in the flexible bearing generates a periodic impact sequence that excites the system impulse response. Due to the rotation of the fault point relative to the stationary sensor and the non-uniform load distribution, the signal undergoes amplitude modulation (AM) arising from the load zone passing effect, time-varying transfer path, and varying measurement angle. Consequently, the vibration signal is modeled as the convolution of an amplitude-modulated impulse train $d\left(t\right)$ with the system impulse response $s\left(t\right)$. When the impulse train is expanded into a Fourier series, the vibration signal can be expressed as

(1) $$\begin{array}{ll} x\left(t\right) & =\left[a\left(t\right)\cdot d\left(t\right)\right]*s\left(t\right)\\ & =\left\{a\left(t\right)\sum_{k=-\infty }^{+\infty }\omega_{f}\exp\left[jk\omega_{f}t\right]\right\}*\left[\exp\left(-\frac{t}{T_{e}}\right)\cos\left(\omega_{n}t\right)\right], \end{array}$$

where $a\left(t\right)$ denotes the AM function, ∗ denotes the convolution operation, $\omega_{f}$ denotes the bearing fault characteristic frequency, $T_{e}$ denotes the time constant of the impulse response decay, and $\omega_{n}$ denotes the natural frequency. Since faults on the inner race, outer race, and rolling elements exhibit distinct kinematic behaviors, the vibration signals induced by these faults are subjected to unique forms of amplitude modulation. Consequently, the amplitude modulation function $a\left(t\right)$ exhibits distinct modulation patterns corresponding to the specific fault location.

### 2.1. Outer Race Localized Fault

Consider a scenario with a localized fault on the outer race. Due to the elliptical wave generator, the radial load is non-uniform and concentrates along the major axis. The fault point periodically passes through the two symmetrical high-load zones while revolving with the outer race. The impact intensity varies with the local contact stress, resulting in amplitude modulation at frequency $2(\omega_{i}-\omega_{o})$.

Simultaneously, the vibration transmission path length and the measurement angle (relative to the fixed sensor) vary periodically at the outer race rotational frequency $\omega_{o}$. Consequently, the outer race fault impulse train is subjected to multiple amplitude modulations. Substituting the product of these modulation functions into (1), the vibration signal model induced by the outer race fault can be represented as

(2) $$\begin{array}{ll} x\left(t\right) & =\left\{\left[1+A_{l}\cos\left(2\omega_{i}t-2\omega_{o}t\right)\right]\left[1+A_{p1}\cos\left(2\omega_{o}t-2\omega_{i}t\right)+A_{p2}\cos\left(2\omega_{i}t\right)\right]\right.\\ & \left.\times \left[1+A_{a}\cos\left(\omega_{o}t\right)\right]\sum_{k=-\infty }^{+\infty }\omega_{\mathrm{of}}\exp\left(jk\omega_{\mathrm{of}}t\right)\right\}*\left[\exp\left(-\frac{t}{T_{e}}\right)\cos\left(\omega_{n}t\right)\right], \end{array}$$

where $\omega_{\mathrm{of}}$ denotes the outer race fault characteristic frequency, $A_{l}$, $A_{p1}$, $A_{p2}$, $A_{a}$ denote constant modulation coefficients of the load zone passing effect, time-varying transfer path, and varying measurement angle, respectively.

### 2.2. Rolling Element Localized Fault

Similar to the outer race fault, the impacts induced by a rolling element fault are similarly subjected to AM effects arising from the load zone passing effect, the time-varying transfer path, and the time-varying measurement angle. However, the faulty rolling element revolves around the axis with the cage at frequency $\omega_{c}$. Consequently, the modulation frequencies are governed by the cage speed, and the vibration signal model can be expressed as

(3) $$\begin{array}{ll} x\left(t\right) & =\left\{\left[1+A_{l}\cos\left(2\omega_{i}t-2\omega_{c}t\right)\right]\left[1+A_{p1}\cos\left(2\omega_{c}t-2\omega_{i}t\right)+A_{p2}\cos\left(2\omega_{i}t\right)\right]\right.\\ & \left.\times \left[1+A_{a}\cos\left(\omega_{c}t\right)\right]\sum_{k=-\infty }^{+\infty }\omega_{\mathrm{rf}1}\exp\left(jk\omega_{\mathrm{rf}1}t\right)\right\}*\left[\exp(-\frac{t}{T_{e}})\cos(\omega_{n}t)\right], \end{array}$$

where $\omega_{\mathrm{rf}1}$ denotes the rolling element fault characteristic frequency.

### 2.3. Inner Race Localized Fault

For a localized fault on the inner race, the fault point rotates synchronously with the wave generator at frequency $\omega_{i}$. Since the inner race remains stationary relative to the wave generator elliptical profile, the angular position of the fault point within the load distribution is fixed. Consequently, the contact stress at the fault point remains constant.

Regarding the transfer path, the relative distance from the fault to the flexspline–circular spline meshing points is constant. However, the transmission distance from the rotating meshing points to the stationary sensor varies periodically, and this path variation introduces an AM effect at $2\omega_{i}$. Simultaneously, the time-varying measurement angle alters at the rotational frequency $\omega_{i}$ as the fault point revolves relative to the fixed sensor. Therefore, the vibration signal model can be expressed as

(4) $$x\left(t\right)=\left\{\left[1+A_{p}\cos\left(2\omega_{i}t\right)\right]\left[1+A_{a}\cos\left(\omega_{i}t\right)\right]\sum_{k=-\infty }^{+\infty }\omega_{\mathrm{if}}\exp\left(jk\omega_{\mathrm{if}}t\right)\right\}*\left[\exp(-\frac{t}{T_{e}})\cos(\omega_{n}t)\right],$$

where $\omega_{\mathrm{if}}$ denotes the inner race fault characteristic frequency.

## 3. Vibration Characteristics Analysis of Robot Joints and Interference Analysis

In practice, vibration signals acquired from robot joints differ substantially from the ideal models. This difference arises mainly from the non-stationary operating conditions and complex multi-source interference inherent to actual robots.

### 3.1. Non-Stationary Modulation

Unlike traditional rotating machinery, robot joints must execute complex trajectories involving frequent acceleration, deceleration, and reversals. This results in time-varying speeds on the harmonic drive, making the fault-induced vibration signals highly non-stationary. Specifically, the fault characteristic frequency varies linearly with the instantaneous shaft speed, and the instantaneous phase is determined by the time integral of the rotational speed. Accordingly, the fault signal under variable-speed conditions can be modeled as

(5) $$x(t)=\left\{A\left[\int \omega_{i}\left(t\right)dt\right]\sum_{k=-\infty }^{+\infty }\exp\left[\int jk\omega_{f}\left(t\right)dt\right]\right\}*\left[\exp(-\frac{t}{T_{e}})\cos(\omega_{n}t)\right],$$

where $\omega_{i}\left(t\right)$ denotes the instantaneous angular velocity of the input shaft. The integral term $\int \omega_{i}\left(t\right)dt$ represents the angular position phase, indicating that the AM function $A\left(\cdot \right)$ is synchronized with the shaft rotation rather than time. $\omega_{f}\left(t\right)$ is the time-varying fault characteristic frequency, which maintains a fixed ratio with the input speed.

This frequency modulation phenomenon leads to uneven spacing of fault pulses in the time domain, which causes severe spectral smearing in the frequency domain. When traditional Fourier spectrum analysis is applied, the fault energy disperses over a wide frequency band, rather than concentrating at a distinct peak. As a result, frequency domain peak searching methods become ineffective for diagnosis.

### 3.2. Multi-Source Interference Mechanism

Vibration signals acquired from robot joints are contaminated by complex background noise originating from sensor motion kinematics, synchronous vibrations induced by manufacturing tolerances, misalignment, and elastic deformation of flexible components.

First, motion-induced interference poses a significant challenge. A sensor mounted on a moving joint measures not only the desired structural vibration but also rigid-body motion components, specifically centripetal and tangential accelerations. In serial robots, this interference is not isolated but is a coupled result of the kinematics of all upstream joints. By utilizing the robot velocity Jacobian matrix $J\left(\theta \right)$, the kinematic acceleration generated in the base frame can be derived and then projected back to the sensor local frame via the rotation matrix R0s. The resulting motion-induced acceleration interference $S_{m}$ is

(6) Smθ,ω,α=R0sθJθα+J˙θ,ωω,

where θ, ω and α represent the vectors of joint angles, angular velocities and accelerations, respectively.

Furthermore, the signal is complicated by inherent mechanical vibrations and coupling effects. Even in a healthy state, manufacturing tolerances and mass imbalance induce strong vibrations that typically overwhelm subtle fault signatures. This complexity is heightened by environmental noise and structural coupling. Specifically, electromagnetic interference from pulse width modulation (PWM) of the motor driver introduces electrical harmonics related to motor pole pairs [24]. Moreover, cross-joint structural coupling allows vibrations to propagate through rigid links, causing sensors to capture asynchronous, uncorrelated noise transmitted from adjacent joints.

**Figure 2:**
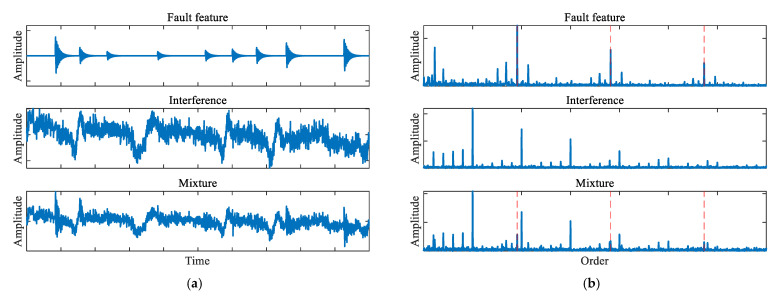
Comparison of the pure fault signal, total interference, and the polluted mixture: (**a**) time-domain waveforms, (**b**) envelope order spectra, where the red dashed lines indicate the fault characteristic order and its harmonics.

Crucially, these multi-source interferences often overlap with the fault features. The spectrum is typically dominated by components originating from manufacturing tolerances, imbalance, and motor harmonics. As a result, the fault features are masked by these interference peaks, as illustrated in Figure 2.

Based on the comprehensive analysis of the fault analytical signal model, time-varying conditions, sensor kinematics, and environmental noise, the raw vibration signal $x_{s}\left(t\right)$ acquired from the robot joint can be modeled as a superposition of the modulated fault signature, deterministic interferences $r\left(t\right)$, and stochastic noise $n\left(t\right)$,

(7) $$x_{s}\left(t\right)=\left\{a\left[\varphi \left(t\right)\right]d\left[\varphi \left(t\right)\right]\right\}*s\left(t\right)+r\left(t\right)+n\left(t\right).$$

where $\varphi \left(t\right)$ denotes the instantaneous angular position of the input shaft.

## 4. Fault Feature Extraction Framework Based on ICA

Based on the signal model established in Section 3, the vibration signal acquired from a robot joint is a complex superposition of the target fault signature, motion-induced interference, and multi-source background noise. ICA provides a powerful solution for such BSS problems. However, its direct application faces two fundamental challenges: non-stationarity induced by time-varying speed and fault features distributed across distinct frequency bands due to channel-dependent transmission paths. To address these challenges, angular resampling and envelope extraction are introduced as preprocessing steps, as detailed in the following subsections.

### 4.1. Angular Resampling

As analyzed in Section 3, the time-varying speed of the robot joint introduces non-periodic frequency modulation to the fault signal, resulting in spectral smearing in Fourier analysis. To resolve this, angular resampling is employed to transform the non-stationary time-domain signal into the stationary angular domain [25].

By utilizing the rotation angle φ(t) of the target joint as a reference, the vibration signal x(t) is resampled at constant angular increments Δφ. In the angular domain, the signal can be expressed as

(8) $$x_{s}\left(\varphi \right)=\left[a\left(\varphi \right)d\left(\varphi \right)\right]*\tilde{s}\left(\varphi \right)+\tilde{r}\left(\varphi \right)+\tilde{n}\left(\varphi \right).$$

Through angular resampling, signal components synchronized with the reference shaft are converted into strictly periodic functions with constant periods in the angular domain. Consequently, the smeared fault energy is refocused into discrete peaks in the order spectrum.

However, components non-synchronous with the target joint speed (e.g., cross-joint coupling, structural resonances, or fixed-frequency electromagnetic noise) are mapped into the angular domain non-linearly. The angular periodicity of these interferences is destroyed, and their energy is smeared across the order spectrum, manifesting as broadband background noise [26]. Furthermore, the structural resonance frequency $\omega_{n}$ is an inherent property of the system and does not vary with speed. In the angular domain, this constant frequency becomes non-stationary. This implies that, while the periodicity of fault impulses is stabilized, the carrier waveform becomes distorted. Therefore, although angular resampling enhances periodicity, the low signal-to-noise ratio caused by source mixing persists, necessitating further separation.

### 4.2. Envelope Extraction

As derived in (1), in a multi-channel measurement scenario, different transmission paths possess distinct impulse response functions, causing the fault-related components to be modulated into disparate frequency bands across channels. However, standard ICA relies on an instantaneous mixing model, which requires the target source to share identical frequency characteristics across all observation channels. Consequently, standard ICA will misinterpret these channel-specific fault manifestations as distinct and independent source components.

Envelope extraction is introduced as a preprocessing step for ICA to address this limitation. By applying the Hilbert transform, the envelope signal removes the effect of the resonance carrier. In the signal envelope, the relationship between the source and the observation approximates a linear, instantaneous scaling suitable for standard ICA.

The convolution of the amplitude-modulated fault impulse train with the system impulse response can be approximated physically. The process amounts to: (1) triggering a system resonance s(t) at each fault occurrence time $t_{k}$; (2) scaling the magnitude by the AM function a(t). Therefore, the signal envelope can be expressed as

(9) $$x_{a}\left(t\right)=\left\{\int \omega_{i}\left(t\right)dt\sum_{k=-\infty }^{+\infty }\exp\left[\int jk\omega_{f}\left(t\right)dt\right]\right\}*\exp\left(-\frac{t}{T_{e}}\right).$$

Equation (9) reveals that the signal envelope only contains the fault characteristics and is not affected by the resonance carrier, thus being applicable to ICA.

### 4.3. Independent Component Analysis

ICA is a statistical technique used to separate independent source signals from linear mixtures. In the specific context of robot joint fault diagnosis, the fundamental model assumes that the observed multi-channel signals X are linear combinations of unknown mutually independent source signals S, expressed as

(10) X=AS+N,

where $X={\left[x_{1},x_{2},\ldots ,x_{m}\right]}^{T}$ represents the observation matrix constructed from a multi-channel vibration signal. Since the sensors rotate with the joint or are distributed across the structure, different vibration sources project into these channels with varying coupling coefficients depending on the transmission path direction, satisfying the multi-channel requirement. The source matrix $S={\left[s_{1},s_{2},\ldots ,s_{n}\right]}^{T}$ contains distinct physical vibration sources, where one component corresponds to the target fault vibration, while others represent interferences. A is the unknown m×n mixing matrix, and N represents additive noise.

The objective of ICA is to estimate a separation matrix W to recover the source $Y=WX\approx \hat{S}$. Algorithms such as Fast-ICA achieve this by maximizing the non-Gaussianity of the output components Y. By doing so, the algorithm effectively isolates the fault impulses from the background noise.

### 4.4. Analysis Procedure

**Figure 3:**
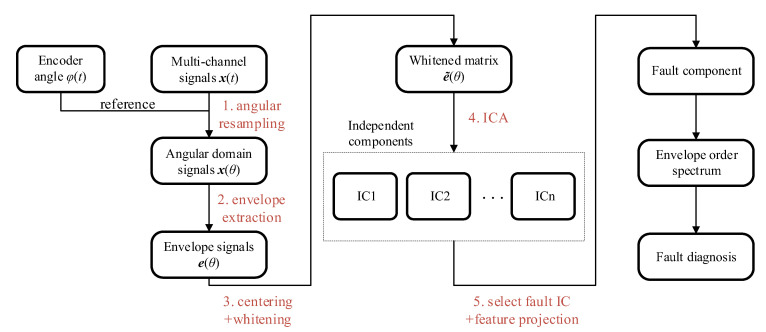
Analysis flowchart of the proposed multi-channel vibration analysis framework.

Based on the above analysis, the complete diagnostic procedure is illustrated in Figure 3 and consists of the following steps:

1. Angular resampling. The raw multi-channel vibration signals $x\left(t\right)={\left[x_{1}\left(t\right),x_{2}\left(t\right),\ldots ,x_{m}\left(t\right)\right]}^{T}$ are resampled at constant angular intervals Δθ using the joint rotation angle signal. This yields the angular domain signals $x\left(\varphi \right)$, restoring the periodicity of fault impacts.
2. Envelope extraction. The Hilbert transform is applied to each resampled channel to obtain the envelope signals $E\left(\varphi \right)={\left[E_{1}\left(\varphi \right),E_{2}\left(\varphi \right),\ldots ,E_{m}\left(\varphi \right)\right]}^{T}$.
3. Preprocessing. The signal envelopes are centered and whitened. Principal component analysis is used to determine the number of principal sources based on the singular value distribution, filtering out minor noise components.
4. Blind source separation. The Fast-ICA algorithm is applied to the whitened envelope matrix. The algorithm iteratively updates the unmixing matrix W to maximize the negentropy of the output components $y_{i}\left(\varphi \right)$. This results in a set of ICs, where one IC is expected to capture the fault signature.
5. Feature projection. ICA has inherent amplitude ambiguity. The contribution of ICs is projected back to the original measurement space using the inverse of the unmixing matrix, recovering the physical amplitude of the fault component and resolving the scaling ambiguity inherent in BSS.
6. Envelope order spectrum diagnosis. Finally, the Fourier transform is applied to the projected fault component to obtain the envelope order spectrum. The health condition of the flexible bearing is diagnosed by examining the magnitude of the spectral peaks at the theoretical fault characteristic orders.

## 5. Experimental Validation

To further validate the effectiveness of the proposed solution, experimental verification is conducted on an industrial robot joint.

### 5.1. Experimental Setting

**Figure 4:**
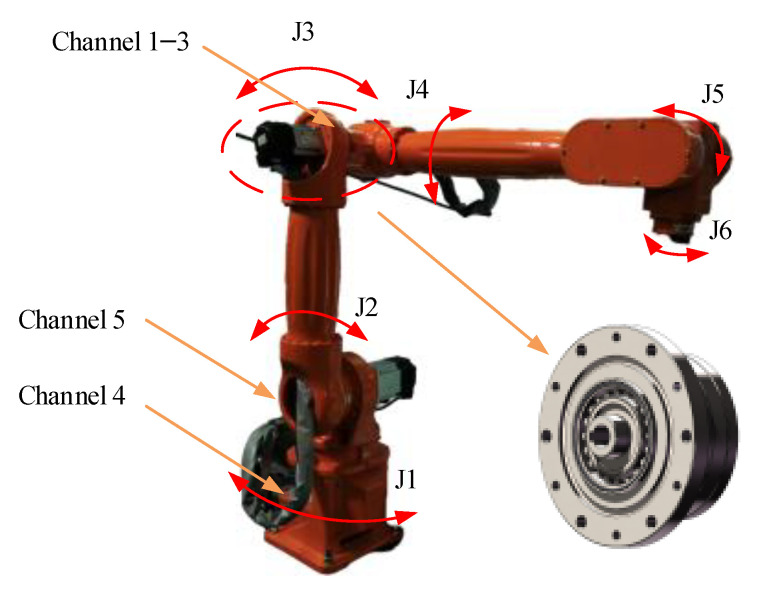
Industrial robot test rig. Orange arrows are used to indicate the sensor mounting locations and the harmonic drive mounting location.

The experimental setup, as shown in Figure 4, consists of a six-degree-of-freedom industrial robot. The diagnosis target is the harmonic drive on Joint 3 (J3), which is driven by a 400 W servo motor. The key parameters of the harmonic drive and its fault characteristic orders are listed in Table 1 and Table 2, respectively.

**Figure 5:**
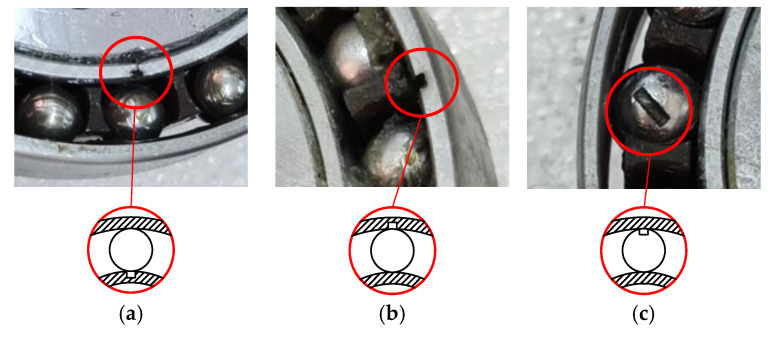
Close-up view of localized faults in harmonic drive: (**a**) inner race, (**b**) outer race, (**c**) rolling element.

To simulate different fault conditions, artificial slots (0.8 mm × 0.8 mm) are machined on the wave generator bearing components. Figure 5 illustrates the faulty components used in the experiments. The experiment consists of four health conditions: (1) baseline, when all components are in a healthy state; (2) inner race fault, when a localized fault is present on the inner race; (3) rolling element fault, when a localized fault is present on a rolling element; and (4) outer race fault, when a localized fault is present on the outer race.

To characterize multi-source vibrations, a five-channel distributed sensor configuration was employed. A triaxial accelerometer was mounted on the Joint 3 casing (Channels 1–3), as illustrated in Figure 4. Reference sensors were installed at the robot base (Channel 4) and the adjacent Joint 2 (Channel 5) to monitor external disturbances and coupled structural vibrations, respectively. A 2500-line encoder recorded the J3 angular position. Vibration signals and encoder signals are sampled at 20,480 Hz and 204,800 Hz, respectively, for a time duration of 200 s.

**Figure 6:**
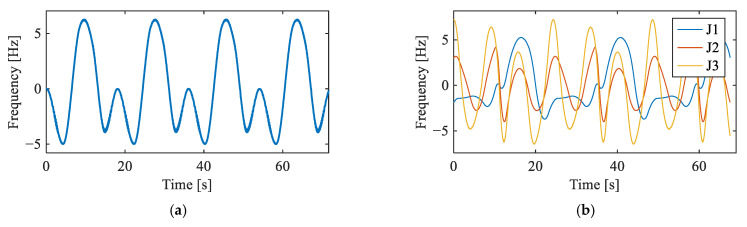
Input shaft rotational frequency: (**a**) J3 in single-joint trajectory, and (**b**) J1, J2, and J3 in multi-joint trajectory.

Two motion trajectories were employed to validate the proposed framework under different operating conditions. In the single-joint trajectory, only J3 was actuated, and five vibration channels were acquired. In the multi-joint trajectory, J1, J2, and J3 operated simultaneously, introducing additional coupled interference from adjacent joints. The rotational speed profiles of the input shafts under both trajectories are provided in Figure 6. For each fault condition under each trajectory, one experimental trial was conducted.

To evaluate the performance of the proposed framework, three baseline methods are employed for comparison: the raw order spectrum, the envelope order spectrum, and a CEEMDAN-based envelope order spectrum [27]. The raw order spectrum is obtained by applying the Fourier transform directly to the angularly resampled signal. The envelope order spectrum is obtained by applying the envelope extraction followed by the Fourier transform. The CEEMDAN-based method decomposes the signal into intrinsic mode functions, from which the fault-related component is selected and analyzed via an envelope order spectrum.

### 5.2. Single-Joint Validation

#### 5.2.1. Baseline Signal Analysis

**Figure 7:**
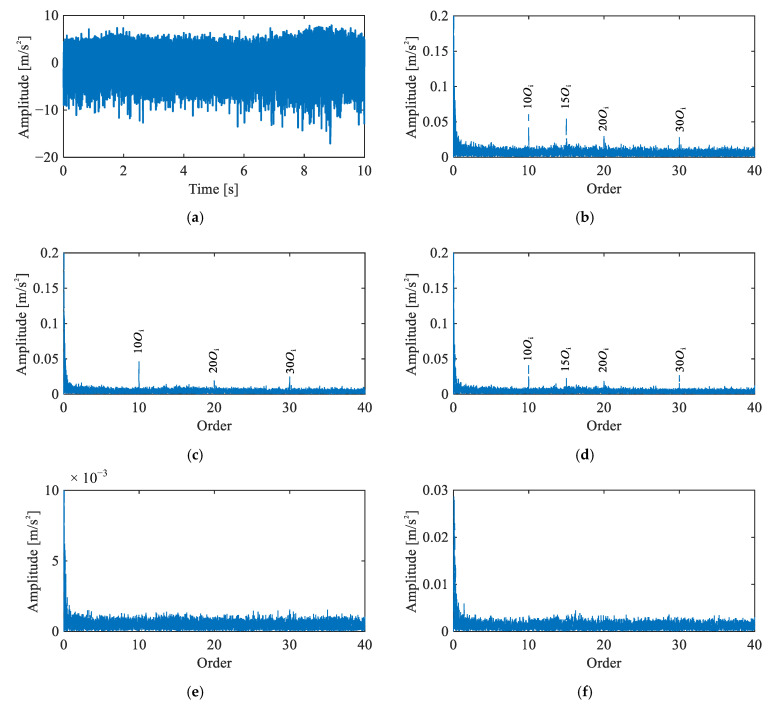
Vibration signals under baseline condition: (**a**) representative time-domain waveform (Channel 2), (**b**–**f**) envelope order spectra of Channels 1–5.

Figure 7 displays the envelope order spectra of the vibration signals from all channels under the baseline condition. In Channels 1–3 (triaxial accelerometer on the target joint), prominent peaks are observed at the 5th harmonic of the input shaft rotational order and its multiples, specifically at $10O_{i}$, $15O_{i}$, $20O_{i}$, and $30O_{i}$. These peaks correspond to the product of the motor pole pairs and the input shaft rotational order harmonics, indicating the presence of electromagnetic vibration from the servo motor. In contrast, Channels 4 and 5 exhibit no significant peaks. Crucially, no peaks corresponding to the fault characteristic orders of the flexible bearing are observed in the spectra. These observations confirm a healthy baseline state, with no fault-induced spectral activity present.

#### 5.2.2. Rolling Element Fault Signal Analysis

**Figure 8:**
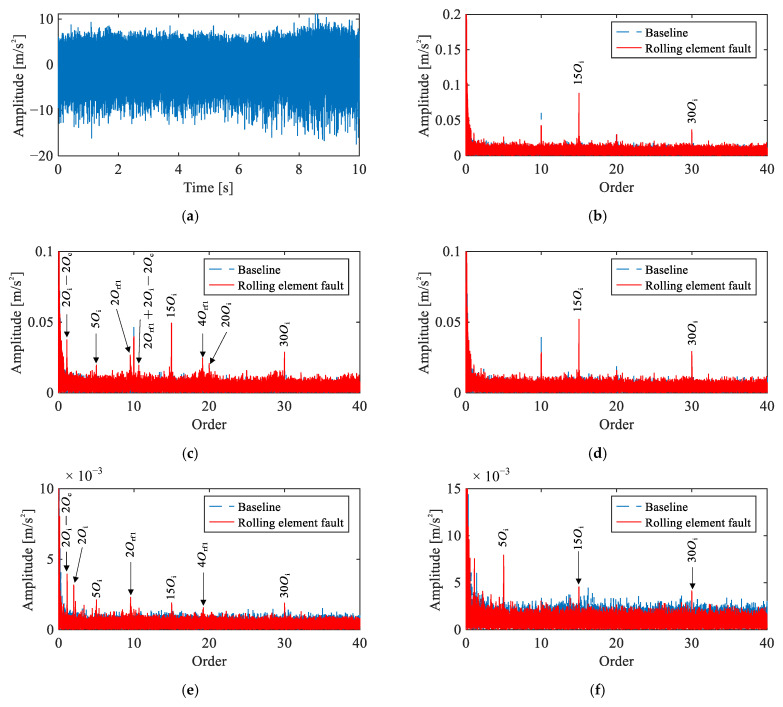
Vibration signals under rolling element fault condition: (**a**) representative time-domain waveform (Channel 2), (**b**–**f**) envelope order spectra of Channels 1–5.

Figure 8 presents the time-domain waveform and envelope order spectra of the Channel 2 vibration signal under the single-joint rolling element fault condition. No distinct impulsive components are observable in the time-domain waveform. In the envelope order spectra, although weak peaks at $2O_{\mathrm{rf}1}$ and $4O_{\mathrm{rf}1}$ are discernible in Channels 2 and 4, the spectra are dominated by motor electromagnetic harmonics at $5O_{i}$ and higher orders, rendering the fault features unidentifiable.

**Figure 9:**
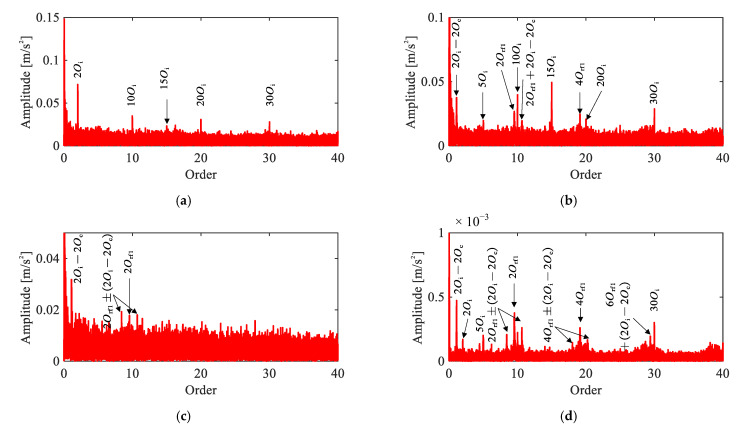
Comparison of signal analysis results for rolling element fault under single-joint operating condition: (**a**) raw signal order spectrum, (**b**) envelope order spectrum, (**c**) envelope order spectrum of CEEMDAN-extracted IMF, (**d**) envelope order spectrum of extracted IC by the proposed method.

Figure 9 presents a comparison of four methods applied to the same signal. In the raw order spectrum (Figure 9a), only harmonics of $5O_{i}$ are observed. The envelope order spectrum (Figure 9b) reveals $2O_{\mathrm{rf}1}$ and $4O_{\mathrm{rf}1}$, but these peaks nearly overlap with $10O_{i}$, making attribution ambiguous. The CEEMDAN-based result (Figure 9c) shows $2O_{\mathrm{rf}1}$ and its sidebands $2O_{\mathrm{rf}1}\pm (2O_{i}-2O_{c})$, yet the peaks exhibit only marginal magnitude above the noise floor. Across all three methods, reliable identification of the rolling element fault remains elusive.

In contrast, the proposed method (Figure 9d) yields clear peaks at even harmonics $2O_{\mathrm{rf}1}$, $4O_{\mathrm{rf}1}$, $6O_{\mathrm{rf}1}$ along with their modulation sidebands at intervals of $2O_{i}-2O_{c}$. The recovery of this characteristic even-harmonic pattern with corresponding modulation sidebands demonstrates the effectiveness of the proposed framework.

#### 5.2.3. Outer Race Fault Signal Analysis

**Figure 10:**
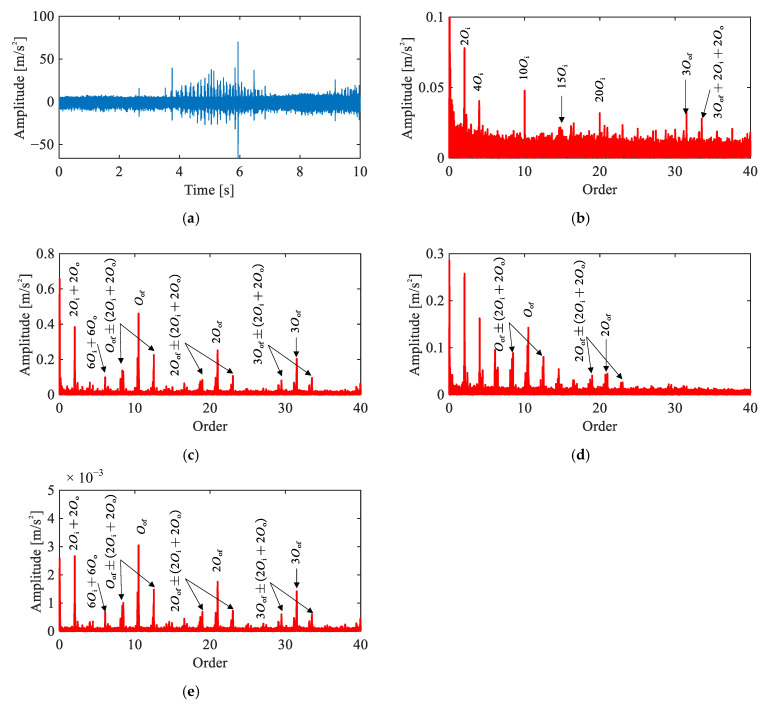
Comparison of signal analysis results for outer race fault under single-joint operating condition: (**a**) representative time-domain waveform, (**b**) raw signal order spectrum, (**c**) envelope order spectrum, (**d**) envelope order spectrum of CEEMDAN-extracted IMF, (**e**) envelope order spectrum of extracted IC by the proposed method.

Figure 10 presents the time-domain waveform and diagnostic results of four methods for the outer race fault signal under the single-joint condition. The time-domain waveform (Figure 10a) exhibits periodic impulsive events with evident time-varying amplitude. This suggests the presence of a localized fault, though precise fault type identification requires further order spectrum analysis.

In the raw order spectrum (Figure 10b), peaks are observed at $3O_{\mathrm{of}}$ and combination components at $3O_{\mathrm{of}}+2O_{i}+2O_{o}$, though the spectrum remains dominated by motor-related harmonics. The envelope order spectrum (Figure 10c) reveals a much clearer fault signature, including $\left(1\sim 3\right)O_{\mathrm{of}}$ and a distinct pattern of modulation sidebands spaced at $2O_{i}+2O_{o}$, i.e., $nO_{\mathrm{of}}\pm \left(2O_{i}+2O_{o}\right)$. The CEEMDAN-based result (Figure 10d) shows $O_{\mathrm{of}}$ and $2O_{\mathrm{of}}$, but the harmonic magnitudes are weaker than those in the envelope order spectrum, suggesting that the decomposition does not fully preserve the fault-related energy.

The proposed method (Figure 10e) yields result closely consistent with the envelope order spectrum. Nevertheless, the fault characteristic peaks exhibit a marginally higher magnitude relative to the noise floor compared to the envelope order spectrum, as quantitatively confirmed in Section 5.4. These spectral features collectively confirm a localized outer race fault.

#### 5.2.4. Inner Race Fault Signal Analysis

**Figure 11:**
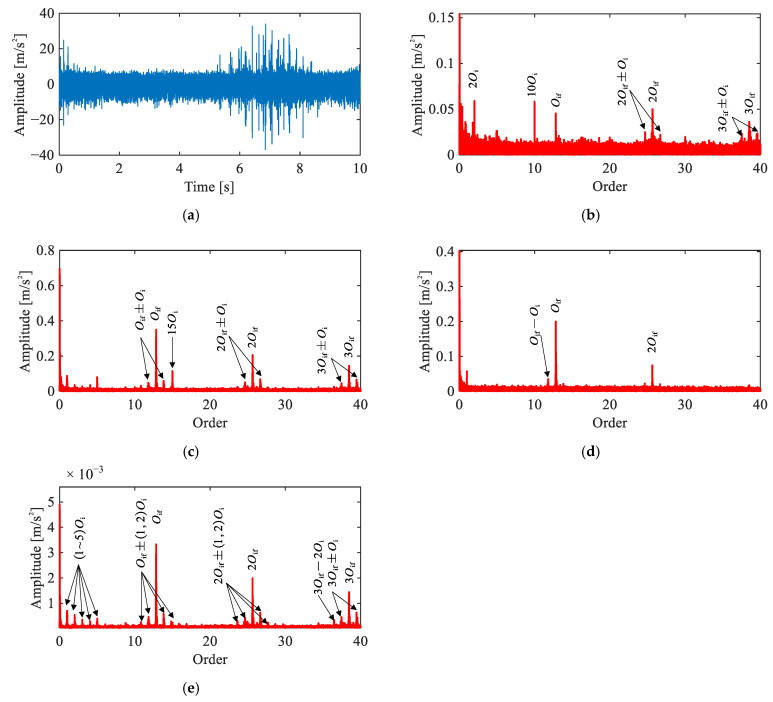
Comparison of signal analysis results for inner race fault under single-joint operating condition: (**a**) representative time-domain waveform, (**b**) raw signal order spectrum, (**c**) envelope order spectrum, (**d**) envelope order spectrum of CEEMDAN-extracted IMF, (**e**) envelope order spectrum of extracted IC by the proposed method.

Figure 11 presents the time-domain waveform and diagnostic results of four methods for the inner race fault signal under the single-joint condition. Similar to the outer race case, the time-domain waveform (Figure 11a) exhibits distinct impulsive events with time-varying amplitude, suggesting the presence of a localized fault.

In the raw order spectrum (Figure 11b), peaks at $O_{\mathrm{if}}$, $2O_{\mathrm{if}}$, and $3O_{\mathrm{if}}$ are observable alongside the motor-related harmonics, suggesting that the inner race fault features are relatively pronounced even in the unprocessed signal. The envelope order spectrum (Figure 11c) further enhances the fault-related orders. However, motor interference such as $15O_{i}$ remains present and overlaps with the fault signature region. The CEEMDAN-based result (Figure 11d) clearly reveals $O_{\mathrm{if}}$, $2O_{\mathrm{if}}$, and their surrounding sidebands, indicating effective separation of the fault component, though higher-order harmonics are not well represented.

The proposed method (Figure 11e) yields prominent peaks at $O_{\mathrm{if}}$, $2O_{\mathrm{if}}$, and $3O_{\mathrm{if}}$ and their sidebands at $nO_{\mathrm{if}}\pm kO_{i}$ (where k=1,2), free of motor-related interference. Compared to the envelope order spectrum, the motor harmonics are effectively suppressed; compared to the CEEMDAN result, a substantially richer harmonic and sideband structure is preserved. These results confirm a localized inner race fault, demonstrating that the proposed method effectively removes interference while retaining comprehensive fault-related information.

### 5.3. Multi-Joint Validation

#### 5.3.1. Baseline Signal Analysis

**Figure 12:**
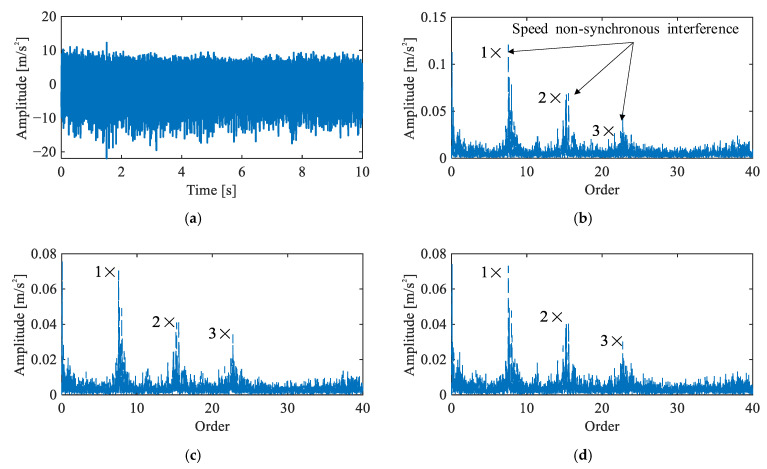
Vibration signals under multi-joint baseline condition: (**a**) representative time-domain waveform, (**b**–**d**) envelope order spectra of Channels 1–3.

Figure 12 presents the time-domain waveform and envelope order spectra of the triaxial vibration signals under the multi-joint baseline condition. Prominent peaks are observed at orders 7.57, 15.15, and 22.73 across all three channels. These peaks form a distinct harmonic structure that does not correspond to any characteristic orders of the J3 harmonic drive and exhibit relatively wide bandwidths, indicating the presence of speed non-synchronous interference originating from the simultaneously operating joints. No fault characteristic orders are identifiable in the spectra. Figure 12 confirms a healthy baseline state under multi-joint operation, while highlighting the additional non-synchronous interference introduced by adjacent joints compared to the single-joint case.

#### 5.3.2. Rolling Element Fault Signal Analysis

**Figure 13:**
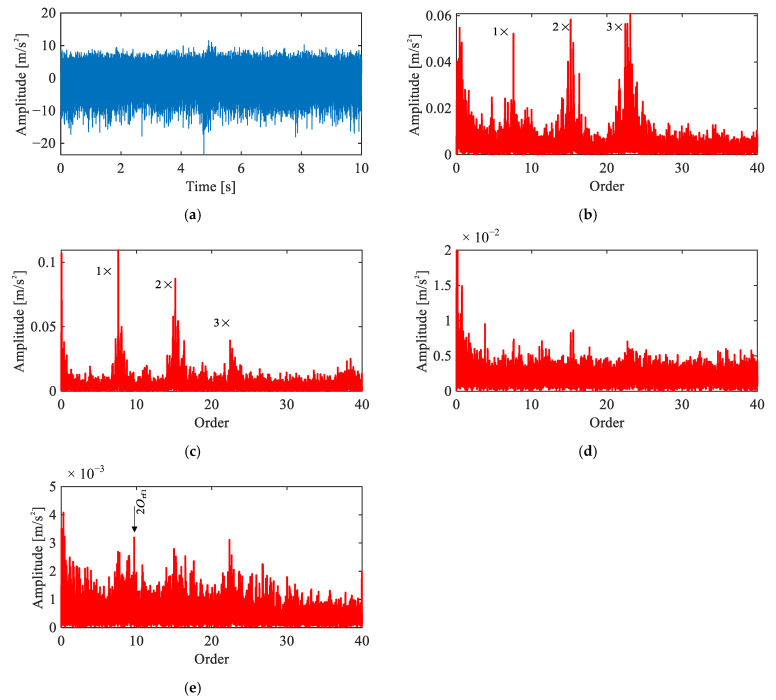
Comparison of signal analysis results for rolling element fault under multi-joint operating condition: (**a**) representative time-domain waveform, (**b**) raw signal order spectrum, (**c**) envelope order spectrum, (**d**) envelope order spectrum of CEEMDAN-extracted IMF, (**e**) envelope order spectrum of extracted IC by the proposed method.

Figure 13 presents the results for the rolling element fault under the multi-joint operating condition. The time-domain waveform (Figure 13a) shows no discernible impulsive features, similar to the baseline, reflecting the severe masking effect of the intensified multi-source interference.

In the raw order spectrum (Figure 13b) and the envelope order spectrum (Figure 13c), the spectra are dominated by the same speed non-synchronous interference clusters observed in the baseline, centered around orders 7.57, 15.15, and 22.73. No components attributable to the J3 harmonic drive or its fault characteristic orders are identifiable. The CEEMDAN-based result (Figure 13d) shows some reduction in interference, but no peaks are identifiable at the theoretical fault characteristic orders.

The proposed method (Figure 13e) recovers a discernible peak at $2O_{\mathrm{rf}1}$, which is absent in the results of all competing methods. Although the feature is relatively weak, this is consistent with the inherently low energy of rolling element faults under intensified multi-joint interference. Its presence at the theoretical fault characteristic order confirms the diagnostic capability of the proposed framework.

#### 5.3.3. Outer Race Fault Signal Analysis

**Figure 14:**
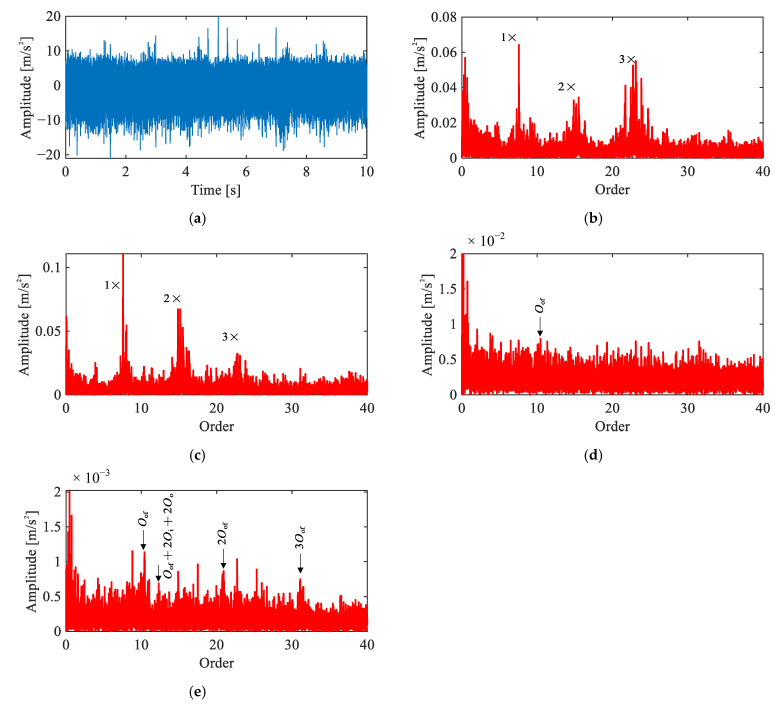
Comparison of signal analysis results for outer race fault under multi-joint operating condition: (**a**) representative time-domain waveform, (**b**) raw signal order spectrum, (**c**) envelope order spectrum, (**d**) envelope order spectrum of CEEMDAN-extracted IMF, (**e**) envelope order spectrum of extracted IC by the proposed method.

Figure 14 presents the diagnostic results for the outer race fault under the multi-joint operating condition. The time-domain waveform (Figure 14a) shows some impulses, though they are not clearly distinguishable from the background interference.

In the raw order spectrum (Figure 14b) and the envelope order spectrum (Figure 14c), the spectra are again dominated by the speed non-synchronous interference clusters, consistent with the baseline, and no fault characteristic orders are identifiable. The CEEMDAN-based result (Figure 14d) reveals a marginal peak at $O_{\mathrm{of}}$, but its magnitude is barely distinguishable from the noise floor.

The proposed method (Figure 14e) yields identifiable peaks at $\left(1\sim 3\right)O_{\mathrm{of}}$. While these features are not as prominent as in the single-joint case, they are substantially clearer than those produced by any of the competing methods. Figure 14 confirms the presence of an outer race fault, with the proposed method providing the most reliable diagnostic result among all compared approaches.

#### 5.3.4. Inner Race Fault Signal Analysis

**Figure 15:**
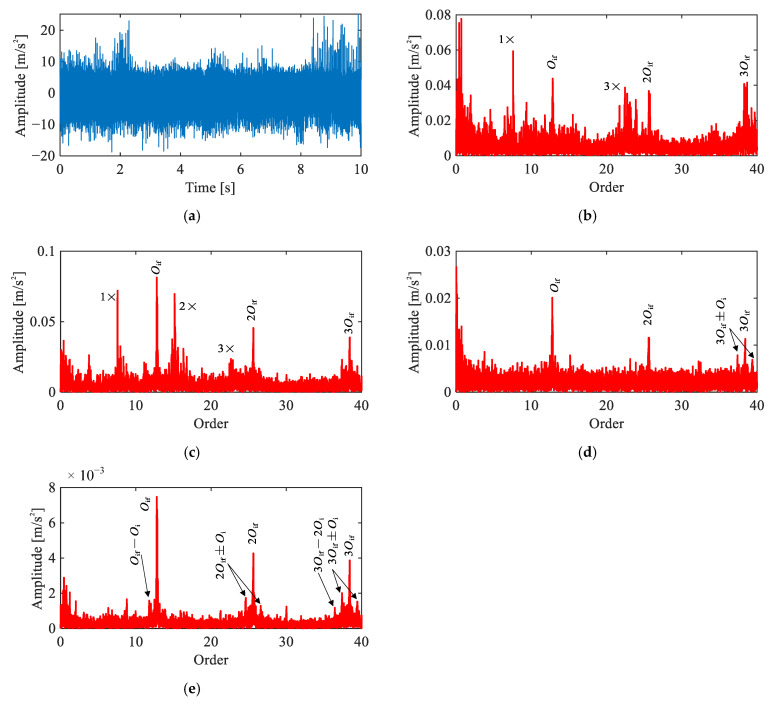
Comparison of signal analysis results for inner race fault under multi-joint operating condition: (**a**) representative time-domain waveform, (**b**) raw signal order spectrum, (**c**) envelope order spectrum, (**d**) envelope order spectrum of CEEMDAN-extracted IMF, (**e**) envelope order spectrum of extracted IC by the proposed method.

Figure 15 presents the diagnostic results for the inner race fault under the multi-joint operating condition. The time-domain waveform (Figure 15a) exhibits more pronounced impulses compared to the other fault types, consistent with the relatively stronger fault signature of inner race faults.

In the raw order spectrum (Figure 15b), both the speed non-synchronous interference clusters and the fault characteristic orders $\left(1\sim 3\right)O_{\mathrm{if}}$ are observable, indicating that the inner race fault features are sufficiently energetic to emerge above the interference even in the unprocessed signal. The envelope order spectrum (Figure 15c) retains some residual interference, but peaks at the fault-related orders are more clearly prominent. The CEEMDAN-based result (Figure 15d) effectively removes the interference, preserving $\left(1\sim 3\right)O_{\mathrm{if}}$ along with sidebands at $3O_{\mathrm{if}}\pm O_{i}$.

The proposed method (Figure 15e) similarly suppresses interference while yielding more prominent fault characteristic orders and a richer sideband structure, including $O_{\mathrm{if}}\pm O_{i}$ and $2O_{\mathrm{if}}\pm O_{i}$. The additional sidebands provide stronger kinematic evidence of the inner race fault mechanism. These results confirm an inner race fault, demonstrating the superiority of the proposed method in both interference suppression and fault feature richness.

### 5.4. Quantitative Comparison

Table 3 summarizes the SNR of fault characteristic orders across all methods and operating conditions. For cases where no identifiable peak is present at a given harmonic, the computed SNR reflects the spectral magnitude at the theoretical order position relative to the background noise floor.

In the single-joint case, the proposed framework achieves the highest SNR across all fault types. For the rolling element fault, the proposed method outperforms the envelope order spectrum by approximately 5 dB and CEEMDAN by over 8 dB at the fundamental harmonic. For the outer race and inner race faults, both the envelope order spectrum and CEEMDAN yield competitive SNR values; however, the proposed method maintains a consistent advantage across all harmonics. This advantage is particularly evident at higher-order harmonics: for instance, the SNR of $3O_{\mathrm{of}}$ drops from 27.00 dB to 9.55 dB with CEEMDAN, compared to 28.10 dB with the proposed method.

In the multi-joint case, all methods show reduced SNR compared to the single-joint case, owing to the intensified coupled interference from simultaneously operating joints. The proposed framework maintains a clear advantage at the lower-order harmonics (2× and 4×) of the rolling element fault. For the higher-order harmonics (6× and 8×), competing methods yield comparable or higher SNR values, but no corresponding peaks are identifiable in their spectral plots, indicating that these values reflect background fluctuations rather than genuine fault signatures. For the outer race and inner race faults under multi-joint conditions, the proposed method similarly maintains the highest SNR across all harmonics.

### 5.5. Discussion

In the single-joint experiments, the proposed framework successfully extracted fault characteristic orders and their modulation sidebands for all three fault types, even under strong electromagnetic interference from the servo motor. In the more challenging multi-joint scenario, speed non-synchronous interference from simultaneously operating joints further obscured the fault signatures. Nevertheless, the framework continued to produce identifiable fault features across all three fault types, demonstrating its robustness under complex operating conditions.

The quantitative comparison in Section 5.4 confirms these observations. The proposed method achieves the highest SNR across all fault types and operating conditions. The advantage is most pronounced for the rolling element fault, where competing methods either fail to produce identifiable peaks or yield SNR values that reflect background fluctuations rather than genuine fault signatures.

Nevertheless, the framework currently relies on manual judgment at two stages: selecting the target independent component and identifying fault peaks in the order spectrum, both of which introduce subjectivity and may be impractical in online monitoring scenarios. Developing automated strategies for IC selection and fault identification remains a direction for future work.

## 6. Conclusions

This paper proposes a multi-channel vibration signal processing framework for fault diagnosis of flexible bearings in harmonic drives of industrial robots. The framework integrates angular resampling, envelope extraction, and ICA to address the dual challenges of variable-speed non-stationarity and strong multi-source interference inherent to robot joint signals. The multi-source interference mechanisms in robot joint vibration signals, including motion-induced kinematic interference and cross-joint coupling, are systematically analyzed to establish the theoretical basis of the framework. Experimental validation under both single-joint and multi-joint operating conditions demonstrates that the proposed framework successfully extracts fault characteristic orders for inner race, outer race, and rolling element faults obscured in the original signals. Quantitative comparison with envelope extraction and CEEMDAN-based methods confirms that the proposed framework achieves higher SNR across all fault types and operating conditions.

While the proposed framework demonstrates promising diagnostic performance, certain aspects warrant further investigation. The framework has been validated under single-fault conditions only; its applicability to compound fault scenarios remains to be established. Future work will focus on developing automated fault identification strategies, including threshold-based peak detection and automated IC selection criteria, as well as extending the validation to compound faults and other robotic systems.

## Figures and Tables

**Table 1:** Industrial Robot J3 Harmonic Drive Configuration.

Parameter	Value
**Number of teeth**	
Circular spline *Z*_c_	162
Flexspline *Z*_f_	160
**Flexible rolling bearing**	
Inner race average radius *R*_i_	24.13 mm
Maximum radial deformation *d*_0_	0.375 mm
Rolling element radius *r*_r_	2.7845 mm
Number of rolling elements *Z*_r_	23

**Table 2:** Characteristic Frequency Orders.

Orders	Value
**Absolute rotating frequency**	
Circular spline	0
Wave generator/input shaft *O*_i_	1
Flexspline/output shaft *O*_o_	0.0125
Cage *O*_c_	0.441
Mesh frequency *O*_m_	162
**Fault characteristic frequency**	
Inner race *O*_if_	12.85
Outer race *O*_of_	10.44
Rolling element *O*_rf1_	4.84

**Table 3:** SNR of fault characteristic orders obtained by each method under single-joint and multi-joint operating conditions.

		Order Spectrum	Envelope Order Spectrum	CEEMDAN + ENVELOPE	Proposed Framework
**Single-joint**	Rolling element	2× 9.22 dB	2× 15.09 dB	2× 11.83 dB	**2**× **20.30 dB**
		4× 7.50 dB	4× 14.38 dB	4× 6.82 dB	**4**× **17.12 dB**
		6× 6.99 dB	6× 10.23 dB	6× 4.13 dB	**6**× **12.54 dB**
		8× 7.04 dB	8× 7.57 dB	8× 3.28 dB	**8**× **11.14 dB**
	Outer race	1× 7.69 dB	1× 34.10 dB	1× 27.80 dB	**1**× **34.76 dB**
		2× 11.43 dB	2× 28.84 dB	2× 17.76 dB	**2**× **29.96 dB**
		3× 14.85 dB	3× 27.00 dB	3× 9.55 dB	**3**× **28.10 dB**
	Inner race	1× 17.84 dB	1× 34.01 dB	1× 31.72 dB	**1**× **35.48 dB**
		2× 18.69 dB	2× 29.39 dB	2× 23.13 dB	**2**× **31.05 dB**
		3× 15.88 dB	3× 26.34 dB	3× 10.67 dB	**3**× **28.23 dB**
**Multi-joint**	Rolling element	2× 8.40 dB	2× 5.75 dB	2× 6.19 dB	**2**× **16.75 dB**
		4× 4.14 dB	4× 7.31 dB	4× 3.10 dB	**4**× **10.23 dB**
		6× 7.71 dB	**6**× **9.66 dB**	6× 4.52 dB	6× 7.22 dB
		8× 1.83 dB	**8**× **7.10 dB**	8× 6.16 dB	8× 5.66 dB
	Outer race	1× 3.93 dB	1× 10.08 dB	1× 10.92 dB	**1**× **16.10 dB**
		2× 10.94 dB	2× 9.49 dB	2× 7.39 dB	**2**× **12.91 dB**
		3× 10.80 dB	3× 9.73 dB	3× 10.51 dB	**3**× **11.90 dB**
	Inner race	1× 18.02 dB	1× 22.51 dB	1× 17.48 dB	**1**× **26.82 dB**
		2× 16.50 dB	2× 19.10 dB	2× 14.61 dB	**2**× **22.85 dB**
		3× 13.53 dB	3× 13.11 dB	3× 8.48 dB	**3**× **16.21 dB**

n× denotes the n-th harmonic of the fault characteristic order. Bold indicates the highest SNR among all methods for each peak in each experiment.

## Data Availability

The raw data supporting the conclusions of this article will be made available by the authors on request.
